# Psychometric evaluation of a screening question for persistent depressive disorder

**DOI:** 10.1186/s12888-019-2100-0

**Published:** 2019-04-23

**Authors:** Elisa Brinkmann, Sarah Glanert, Michael Hüppe, Ana Sofia Moncada Garay, Sophie Tschepe, Ulrich Schweiger, Jan Philipp Klein

**Affiliations:** Department of Psychiatry and Psychotherapy, University of Lübeck, 23562 Lübeck, Germany

**Keywords:** Persistent depressive disorder, Dysthymia, Depressive disorders, Screening question, Rater agreement, Diagnostic interview

## Abstract

**Background:**

About one in five patients with depression experiences a chronic course. Despite the great burden associated with this disease, there is no current screening instrument for Persistent Depressive Disorder (PDD). In the present study, we examine a short screening test, the persistent depression screener (PDS), that we developed for DSM-5 PDD. The PDS is comprised of one question that is administered following an initial self-assessment for depression.

**Methods:**

Ninety patients from an inpatient clinic/day clinic specialized in treating depression completed the PDS. They were also assessed using a structured clinical interview covering the DSM-5 criteria for PDD. Retest reliability was examined after two weeks (*n* = 69, 77%).

**Results:**

In this sample, the prevalence of PDD was 64%. Sensitivity of the PDS was 85% with a positive predictive value of 80%. Specificity was 63%. Positive and negative likelihood ratios were 2.3 and .24, respectively. Agreement between the PDS results and the outcome of the clinical interview was moderate (*Cohen’s Kappa* κ = .48 ([95%-CI .28, .68], *p* < .001, *SE* = 0.10)). Prevalence-adjusted bias-adjusted Kappa was *PABAK* = .53. Retest reliability of the PDS was moderate (*Cohen’s Kappa* κ = .52 ([95%-CI .3, .74], *p* < .001, *SE* = 0.11)).

**Conclusions:**

The present study shows that the PDS - when applied following a self-rating depression scale - might be a valid and reliable way to detect PDD. However, the results of the PDS must be confirmed by a diagnostic interview.

## Background

Worldwide, depression is one of the leading causes of burden of disease [1]. Mortality of people currently affected by depression is considerably higher compared to people not suffering from depression [2]. Lifetime prevalence for a major depressive episode is assumed to be around 17% [3–5]. About one fifth of people suffering from major depression experience relevant symptoms for two years or longer and therefore meet the criteria for persistent depressive disorder (PDD; [6–10]). The longer a person suffers from a depressive disorder, the less likely recovery becomes [11]. In the fifth version of the Diagnostical and Statistical Manual of Mental Disorders (DSM), the American Psychiatric Association (APA) summarized various forms of chronic depression in the section “Persistent Depressive Disorder” (DSM-5; [12]). Compared to patients with episodic forms of depression, patients with PDD have a higher rate of comorbidities and even suicide attempts [3, 9, 13].

The identification and subsequent treatment of depressive disorders, especially chronic forms, is essential as they cause intense suffering for those affected, their families and society as a whole [14]. The U.S. Preventive Service Task Force and its Canadian counterpart recommend screenings for depression provided that adequate treatment is available [15, 16]. Tried and tested screening instruments are available for depressive disorders (e.g. [17]). Very short screening instruments have proven to adequately detect depression, like the Patient Health Questionnaire-2 (PHQ-2; [18, 19]) or the 5-Item World Health Organization Well-Being Index (WHO-5; [20]). Other screening instruments specifically developed for depression were successfully tested in particular sub groups such as pregnant and postpartum women. The U.S. Preventive Service Task Force states that there is a moderate net health benefit in screening this specific population [16]. It is necessary to identify chronic courses of depression since treatment of chronically depressed patients seems to be more successful when their particular needs and deficits, such as interpersonal problems and comorbidity with personality disorders, are directly addressed [21]. Patients with chronic depression seem to respond better to specific forms of therapy, e.g. the cognitive behavioral analysis system of psychotherapy (CBASP), than to unspecified forms of therapy [22, 23].

To our knowledge, no screening for PDD has been developed so far. After protracted forms of depression had been conceptualized as dysthymia in DSM-III, a question that screened for this condition was developed and tested [24]. However, in DSM-III dysthymia is somewhat differently defined than PDD in DSM-5. In particular, PDD can be diagnosed if depressive symptoms have been present almost all of the time (persistent depressive episode). This underlines the need and urgency for an updated screening.

In the present study we examine the persistent depression screener (PDS) – a screening question for DSM-5 Persistent Depressive Disorder – that can be administered following a self-rating scale for depression. The question reads: *“The previous questions covered various symptoms of depression. Now, please consider: When was the last period of two months or longer that you were not impaired by these symptoms?”.* We hypothesize that the PDS has adequate psychometric properties to detect PDD when accompanied by an initial self-assessment of depressive symptomatology. Specifically, we hypothesize that PDS results will at least moderately agree with results of a structured diagnostic interview.

## Methods

### Participants

Participants were recruited at the inpatient/day clinic treatment program for depression at the Department of Psychiatry and Psychotherapy, University of Lübeck, Germany. Participants did not receive financial compensation. The present study uses data from the *ICARE*-Study (*I*nvestigating *C*are Dependency *A*nd its *R*elation to outcom*E*) designed to investigate the German version of the *Care Dependency Questionnaire* [25]*.* The ICARE-study was conducted in accordance with the Declaration of Helsinki and it was approved by the ethics committee of the University of Lübeck. Inclusion and exclusion criteria for the study were modeled on the treatment program’s admission criteria. The treatment program focusses on psychotherapy for depression (mainly CBASP [26] and MCT [27]) and lasts for six weeks. Minimum age for participation in the study was 18 years. An adequate understanding of the German language and informed written consent were required. As we aimed to only include subjects who were not yet familiar with the treatment program, we only accepted patients to the study if it was their first admission to the treatment program. Exclusion criteria were acute suicidality, a history of schizophrenia, delusional disorder, substance use disorder or bipolar disorder as well as a known diagnosis of an acute somatic illness that requires treatment. Only data from patients who completed both the PDS and the clinical interview was analyzed.

### Materials

#### Persistent depression screener (PDS)

We developed the PDS, a paper-and-pencil screening composed of one question. It was administered following a self-rating instrument for depressive symptoms: the Quick Inventory of Depressive Symptomatology (QIDS-SR; [17, 28]). The PDS is based on the DSM-5 criteria for PDD and focusses on criterion C for chronicity of the symptoms (“During the 2-year period of the disturbance, the individual has never been without symptoms … for more than two months at a time”). The translated screening question reads:

“The previous questions covered various symptoms of depression. Now, please consider: When was the last period of two months or longer that you were not impaired by these symptoms?”

The following response options were given:

- less than a year ago.
- more than a year but less than 2 years ago.
- more than 2 years but less than 5 years ago.
- more than 5 years but less than 10 years ago.
- more than 5 years ago.

Answers a) and b) were determined to be indicative of a likely absence of PDD (“PDS negative”). Answers c) to e) indicate a likely presence of PDD (“PDS positive”).

Before we collected the data for the main sample, we conducted a pilot study (*N* = 5) to ensure comprehensibility and feasibility of the PDS. Participants of the pilot study completed the PDS and the interview and these results were examined. The screening outcomes of two participants differed from their interview-based diagnoses. We conducted additional semi-structured interviews with these patients to determine their interpretation of the screening question. Based on this information, we slightly amended the wording of the PDS to improve clarity. The modified and the original question were then presented to the two participants. Both expressed a clear preference for the modified question. As a result, we used the revised PDS to collect data for the main sample. Participants of the pilot study were excluded from all further statistical analyses.

#### Clinical interview for PDD

Trained raters collected diagnostic information on the presence and course of the depressive disorder according to DSM-5 criteria for depressive disorders using a structured interview [29, 30]. The interview was based on the Structured Clinical Interview for DSM (SCID). The order of the questions was changed to increase ease of administration in the diagnosis of PDD (assessment for current depressive episode, past depressive episode and persistent depressive episode; assessment of number of depressive episodes; assessment for presence of dysthymic syndrome; assessment of early versus late onset). Other studies also successfully employed this interview (e.g. [31, 32]). Participants were diagnosed with PDD when meeting DSM-5 criteria for a pure dysthymic syndrome, for a persistent major depressive episode, for persistent depressive disorder with intermittent major depressive episodes, with current episode as well as without current episode. The clinical interview served as the criterion standard for the PDS in this paper.

#### Quick inventory of depressive symptomatology - self report (QIDS-SR)

The German translation of the *Quick Inventory of Depressive Symptomatology - Self Report* was applied prior to the PDS to establish if the patient suffered from depressive symptoms. It is a valid and reliable self-assessment tool of depression severity [28]. It consists of 16 questions concerning depressive symptoms experienced in the last 7 days. The total score ranges from 0 to 27 with higher scores reflecting a greater severity of symptoms [17].

#### Hamilton-Rating-Depression-Scale-6 (HRDS-6)

The *Hamilton-Rating-Depression-Scale-6* is a short (six items) clinician-rated assessment scale for the severity of depressive symptomatology. It is the shortened version of the original scale with 17 items [33]. Symptoms are rated based on the patient’s report and the clinician’s observation with total scores ranging from 0 to 22 [34].

### Procedure

**Fig. 1:**
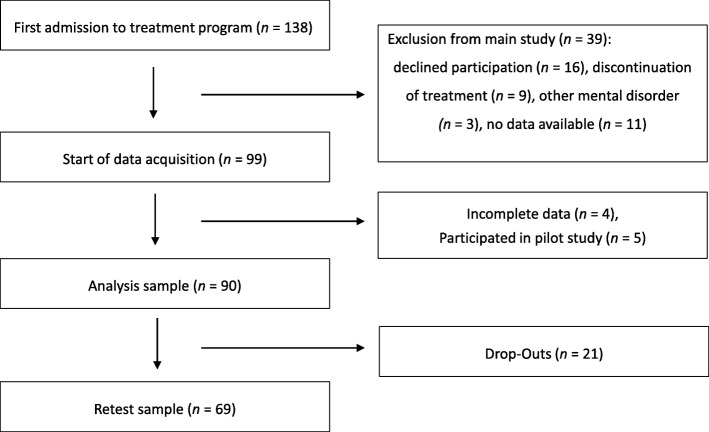
Study Flowchart

Between May 2017 and April 2018 all patients were contacted and informed about the study within the first days of their admission to the treatment program. If patients were eligible for the study (e.g. no discontinuation of treatment, for further information please refer to Fig. 1), they were briefed in detail on the goal and the procedure of the study and had to provide informed consent to participate. Subsequently trained graduate students with a clinical psychology major (EB and ST) conducted the clinical interview and the HRDS-6. We handed out several questionnaires covering demographic information, the QIDS-SR and the PDS to participants within the first week of their treatment. Patients were included in the analysis sample if the questionnaires as well as the interview were completed. Questionnaire data was *anonymized using the examiner’s initials followed by a serial number* (e.g. EB034). After 2 weeks, patients completed another self-assessment of depressive symptoms and the PDS was handed out again to collect data for retest reliability. For detailed information on the procedure of the study, please refer to Fig. 1.

### Statistical analyses

Statistical analyses were conducted using SPSS (IBM SPSS Statistics for Windows, version 22.0). All statistical tests were two-tailed tests with significance levels set at *p* ≤ .05. Standard errors (*SE*) and 95%-confidence intervals (*CI*) are provided in the result section. Missing values were not substituted. To assess the quality of the PDS in a clinical context, we report common measures like sensitivity, specificity, predictive values as well as likelihood ratios. It should be noted that unlike sensitivity, specificity and likelihood ratios, predictive values depend on the prevalence of PDD in the sample [35].

To examine agreement between the PDS result and the diagnosis derived from the clinical interview, *Cohen’s Kappa* (κ) was utilized. κ is a coefficient for rater agreement taking into account chance agreement. Values for κ range between − 1 and + 1, with + 1 indicating total agreement between outcomes [36]. Landis and Koch’s (1977) guideline describes agreement as *poor* at a value of 0, as *slight* when κ = 0–.20, as *fair* when κ = .21–.40, as *moderate* when κ = .41–.60, as *substantial* when κ = .61–.80 and as *almost perfect* when κ = .81–1 [36]. However, Kraemer et al. (2012) state that with DSM-5-diagnoses in a clinical context κ-values ranging between .41 and .60 are *realistic* and values between .21 and .40 are *acceptable* [37].

As a high prevalence of PDD diagnoses was expected in our sample, an alternative calculation of κ was conducted. The *prevalence-adjusted bias-adjusted Kappa* (*PABAK*) takes into account the categorization by the PDS and the prevalence of the disease. A *bias index (BI)* is calculated to check for possibly differing proportions of PDD diagnoses deriving from the clinical interview and the PDS. If the marginal proportions of outcomes are equal, there is no bias between PDS and interview (*BI* = 0). The BI reaches a maximum of 1, when there is no overlap in the instruments’ ratings. A *prevalence index (PI)* is reported to assess the potentially differing relative probability of the two categories *likely diagnosis of PDD* and *unlikely diagnosis of PDD*. *PI* is 0, if both categories are equally likely. If only one of the two categories occurs in this sample, *PI* is +/− 1. A very high probability of one category increases chance agreement between the outcomes of the PDS and the clinical interview. Higher values of chance agreement result in lower κ values. For further information about the calculation of *PABAK,* please refer to Byrt, Bishop and Carlin (1993) [38].

Additionally, Cramér’s *V* (5 × 2 table) is reported as a measure of association between the result of the original (not dichotomized) screening question and the outcome of the corresponding interview question. Different thresholds of the screening answers were also examined to assess the most beneficial proportion of sensitivity and specificity with the *Youden-Index J* (sensitivity + specificity – 1): the higher the value of *J*, the more reliable the test outcome [39].

## Results

### Characteristics of the sample

The analysis sample comprised 90 individuals. For detailed information on participant recruitment, please refer to the study flowchart (Fig. 1). Out of the 90 participants, 18 (20%) were inpatients and 72 (80%) were day clinic patients. Table 1 shows participants’ demographic and clinical characteristics.

**Table 1:** Demographics and clinical characteristics of the analysis sample (*N* = 90)

Demographics	*n* (%)
Sex (female)	46 (51)
Married and cohabiting	24 (27)
Higher secondary school qualifying for university	30 (33)
Employed	51 (57)
Day clinic patients	72 (80)
	mean (SD / range)
Age (years)	41.67 (13.70/19–64)
Use of health care resources in past 12 months	
nights spent as an inpatient in a clinical facility	12.41 (24.05)
visits to a psychiatrist/neurologist	3.82 (5.67)
visits to a psychotherapist	6.35 (9.97)
Diagnoses	*n* (%)
PDD	58 (64)
persistent depressive episode	17 (19)
with intermittent depressive episodes, with current episode	39 (43)
with intermittent depressive episodes without current episode	1 (1)
with pure dysthymic syndrome	1 (1)
Recurrent depressive disorder	27 (30)
with current depressive episode	23 (26)
currently in partial remission	4 (4)
First depressive episode	3 (3)
Other	2 (2)
severe anxiety disorder	1 (1)
recent suicide attempt	1 (1)
Up to five depressive episodes in their lifetime	58 (64)
Early onset of depression (before age of 21)	50 (56)
	mean (SD)
Number of depressive episodes	8.93 (15.83)
Depression severity	
HRDS-6	10.58 (4.44) *
QIDS-SR	15.14 (5.77) *

*Note. SD* standard deviation. * The average severity of depression can be rated as moderate

### Psychometric properties of the PDS

The diagnosis based on the clinical interview concurred with the PDS result in 69 cases (*N* = 90, 77%) as Table 2 illustrates. As shown in Table 2, sensitivity of the PDS was 85%, with 80% accurate positive screening outcomes (*positive predictive value*). Specificity was 63%. The resulting Youden-Index was *J* = .48. There were 16% false-negative and 38% false-positive results. Of the patients with a negative PDS, 69% did in fact not have a diagnosis of PDD (*negative predictive value*). The resulting positive likelihood ratio was 2.3, meaning it was 2.3 times more likely that a subject with PDD had a positive PDS than subjects without PDD having a positive PDS. The negative likelihood ratio was .24, meaning it was 4.2 times more likely that a subject without PDD had a negative PDS compared to subjects with PDD having a negative PDS. Jaeschke et al. (1994) regard values of these magnitudes as *small, but sometimes important* [40].

**Table 2:** Agreement between the diagnoses from the interview and the PDS

		PDD diagnosis based on the clinical interview		total
		present	not present	
likely PDD diagnosis based on PDS result	present	49 (85%)	12 (38%)	61 (68%)
	not present	9 (16%)	20 (63%)	29 (32%)
	total	58	32	90

*Note. PDS* persistent depression screener. *PDD* Persistent depressive disorder. The table shows the agreement (number of subjects, %) between the diagnoses from the clinical interview and the PDS results about chronicity of the depressive symptomatology

*Cohen’s* κ was .48 ([95%-CI .28, .68], *p* < .001, *SE* = 0.10). The strength of agreement can hence be considered *moderate* with a range from *fair* to *substantial* [41]. Bias between PDS results and clinical interviews was negligible for the value of κ (*BI* = .03). The prevalence effect was moderate (*PI* = .32). This moderate prevalence effect implies that *Cohen’s* κ might be an underestimation of the agreement between the PDS and the clinical interview. We therefore calculated the prevalence-adjusted bias-adjusted Kappa (*PABAK*), which was .53. Accordingly, this can be interpreted as a *moderate* agreement between the PDS results and the outcomes of the clinical interviews [41]. When the answers to the PDS were not dichotomized but treated as an ordinal variable for agreement with the interview results, a significant and strong relation of Cramér’s *V* = .59, *p* < .001 was determined. In this sample 98% (*n* = 88) of participants suffered from a depressive disorder, the remaining two patients who were not diagnosed with depression were correctly categorized by the PDS as *not likely suffering from PDD*.

When a patient had been suffering from depressive symptoms for more than 2 years, the PDS categorized the patient as having a *likely diagnosis of PDD* (answers [c] to [e]). The threshold for a PDD diagnosis can be shifted to examine its accuracy. Table 3 shows that when examining the Youden-Index there are two possible thresholds in the answers to the PDS – the original one at *more than 2 years* (answers [c] to [e]) and the threshold at *more than 5 years* (answers [d] and [e]). The latter provided a larger Youden-Index of *J* = .56 compared to *J* = .48 of the original threshold. However, it could only offer a sensitivity of 59%, which does not meet the requirements of how a screening instrument for depression should perform [42]. It can be concluded that the original threshold at more than 2 years (answers [c] to [e]) showed the highest agreement coefficient in combination with a high sensitivity and a reasonable specificity. It offered the most accurate and valuable information.

**Table 3:** Diagnostical thresholds of the PDS

Threshold for last period without complaints	κ	*p*	sensitivity	specificity	J
More than 1 year ago (a vs. b-e)	.30	.002	90%	38%	.28
More than 2 years ago (a-b vs. c-e)	.48	< .001	85%	63%	.48
More than 5 years ago (a-c vs. d-e)	.48	< .001	59%	97%	.56
More than 10 years ago (a-d vs. e)	.18	.007	26%	97%	.23

*Note.* PDS persistent depression screener. The table shows the calculated value of Kappa (K) with the associated level of significance (*p*), sensitivity, specificity and the resulting Youden-Index (*J*) for varying diagnostical thresholds of the PDS

To examine the understanding of the PDS, we tested for differences in the agreement between outcomes of the interview and outcomes of the PDS by controlling for level of education. We found a slightly better value for *Cohen’s* κ for patients with a higher level of education (κ = .51 ([95%-CI .20, .82], *p* < .005, *SE* = 0.16, *n* = 30) compared to patients with lower education (κ = .46 ([95%-CI .22, .70], *p* < .001, *SE* = 0.12, *n* = 59). Both values can be interpreted as *moderate* [41]. Sensitivity and specificity of the PDS were 83 and 67% for patients with higher education, whereas sensitivity for patients with lower education was 85% and specificity was 60%.

### Retest reliability of the PDS

Data was collected again from 69 participants after an interval of 2 weeks to determine retest reliability (77% of main analysis sample). Agreement between the first result of the PDS and its repetition was 80% (55 of 69 cases). The agreement rate with *Cohen’s* κ = .52 ([95%-CI .3, .74], *p* < .001, *SE* = 0.11) can be interpreted as *moderate* [41]. After adjusting for a small bias (*BI* = −.06) and a moderate prevalence effect (*PI* = .39), the agreement rate was supported by *PABAK* = .59. When the answers of the PDS are not dichotomized, but examined as an ordinal variable for agreement, a moderate to substantial relation was detected, Spearman’s ρ = .49, *p* < .01.

## Discussion

To our knowledge, the present study is the first to examine a screening question for PDD. We showed that a short screening of one item is sufficient to distinguish between cases of PDD and non-PDD when administered after a symptom severity rating for depression in a treatment program for depressive disorders. A good sensitivity and positive predictive value with a reasonable specificity suggest that in this very setting, i.e. an inpatient/day clinic treatment program for depression, the outcome of the PDS is a valid indicator of further diagnostic effort concerning the presence of PDD. The outcomes of the PDS moderately corresponded with the diagnoses stemming from clinical interviews. The range of the κ value can be interpreted as *fair* to *substantial* [36]. As mentioned before other interpretations suggest that in clinical contexts this range is *acceptable* to *realistic* [37]. The prevalence-adjusted bias-adjusted Kappa (*PABAK*) value supports our findings. Therefore, we are satisfied with the results.

The agreement between the screening results and the repetition 2 weeks later was moderate, therefore retest reliability is assessed as good in this specific setting. The value of the corresponding *PABAK* supports this. Twenty-one (23%) out of 90 patients in the main analysis sample differed in their outcomes of the PDS and the interview. The majority of these cases (*n* = 14, 67%) chose a screening answer around the threshold of the diagnostic criterion for PDD (answers [b] or [c]). We believe that the specificity of 63% can be accepted for the PDS. If a patient is categorized as *likely suffering from PDD* by the PDS, this result must be confirmed with a clinical diagnostic interview.

As this study was part of a bigger project, we did not define the sample size for the psychometric assessment of the PDS a priori. According to Sim and Wright (2005) a sample size of *n* = 43 is acceptable for the detection of a coefficient of κ = .50 with a two-tailed test (Null Value of κ = .00) and a test power of 90% [43]. Our sample size (*N* = 90) is therefore adequate to test the main research hypothesis.

With the present instrument being the only screening for PDD, we cannot directly compare it to similar measures. The U.S. Preventive Services Task Force (2009) notes that most depression screenings show a sensitivity between 80 and 90% and a specificity of 70 to 85% [37]. The PDS does not reach that value for specificity but complies with the value for sensitivity. The previously described screening instrument for DSM-III-Dysthymia showed slightly better sensitivities (89–92%) but lower specificities (35–62%) in Mental Health settings [24].

The psychometric properties of the PDS compare favorably to the properties of existing depression screeners, namely the PHQ-2, the Hospital Anxiety and Depression Scale (HADS) and the WHO-5. The PHQ-2 is a short screening for depression, consisting of only two questions. Compared to the PDS, the PHQ-2 showed a slightly better sensitivity of 87% and better specificity of 78% at the chosen cut-off point for the diagnosis of major depression, but a less favorable value of κ of .43 [19]. A series of studies on other screening instruments for depression including the HADS and the WHO-5 showed similar results for sensitivity but somewhat better specificity compared to the PDS [18, 44, 45]. We therefore consider the psychometric properties of our measures to be in an acceptable range.

The PDS was examined in a treatment program specialized in the treatment of depression, so prevalence of PDD was expected to be higher than in other clinical contexts. Predictive values were good but should be interpreted with caution. These measures are dependent on the prevalence of the examined disorder and therefore influenced by the high base rate of PDD diagnoses in this particular sample. The other measures are reliable nevertheless, because sensitivity and specificity as well as likelihood ratios are independent of prevalence. The impact of prevalence on κ was considered and examined by the calculation of *PABAK*.

### Strengths and limitations

Strengths of the current study include that our sample is similar to a representative population sample reporting depressive symptoms in a number of demographic characteristics including age, gender, employment status and notably education (most samples of patients in psychotherapy programs are more highly educated than the general population) [46]. We did not find that education level substantially affected our screening results. Also, we performed a broad range of calculations to psychometrically test the PDS.

Regarding limitations of this study, it should be noted that patients were repeatedly asked about the chronicity of their depression both by clinical staff in the treatment program and research staff. The repeated administration of these questions might have influenced the patients’ answers. Compared to patients treated in a setting less specialized in depression, patients in our study likely had more opportunities to reflect on when they have last felt free from symptoms of depression. This might have inflated the accuracy of our screening test. Finally, the pretest probability for a depressive disorder was comparatively high in this sample given that data was collected in a treatment program specialized in depression.

### Future research

Future studies should examine the practicality of the PDS in different medical contexts with a lower prevalence of depression (e.g. in general psychiatric care). Future studies should also verify whether the screening question could be reworded for use in a clinical intake interview. In this setting clinicians could ask the following question after the assessment of current depressive symptomatology: “When have you last experienced a period of two months or longer when you were not impaired by depressive symptoms?” It has been shown that asking simple questions on depressed mood and anhedonia in a clinical assessment interview can perform similar to longer instruments [47].

## Conclusion

The persistent depression screener (PDS) can be administered economically after an initial severity assessment in patients with depression. It showed good sensitivity and moderate accuracy in comparison to the results of a clinical interview. Its brevity, the limited administration effort and low cost make it an economic instrument to elicit information about chronicity of depression. However, the outcome of the PDS must be confirmed by a diagnostic interview. If our results hold up in future studies, mental health clinics could utilize this screening question to detect PDD and thus provide patients with specific treatment.

## Abbreviations

APA: American Psychiatric Association
CBASP: Cognitive Behavioral Analysis System of Psychotherapy
DSM: Diagnostical and Statistical Manual of Mental Disorders
HADS: Hospital Anxiety and Depression Scale
HRDS-6: Hamilton-Rating-Depression-Scale-6
ICARE: Investigating Care Dependency And its Relation to outcome
MCT: Metacognitive Therapy
PABAK: Prevalence-adjusted bias-adjusted Kappa
PDD: Persistent Depressive Disorder
PDS: Persistent depression screener
PHQ-2: Patient Health Questionnaire-2
QIDS-SR: Quick Inventory of Depressive Symptomatology – Self-Report
SCID: Structured Clinical Interview for DSM
WHO-5: 5-Item World Health Organization Well-Being Index
